# Photoinactivation of the bacteriophage PhiX174 by UVA radiation and visible light in SM buffer and DMEM-F12

**DOI:** 10.1186/s13104-023-06658-8

**Published:** 2024-01-02

**Authors:** Florian Sommerfeld, Laura Weyersberg, Petra Vatter, Martin Hessling

**Affiliations:** Department of Medical Engineering and Mechatronics, Ulm University of Applied Sciences, Albert-Einstein-Allee 55, D-89081 Ulm, Germany

**Keywords:** PhiX174, UVA, Visible irradiation, Radiation inactivation, Blue light, Violet light, External photosensitizers, Riboflavin

## Abstract

**Objective:**

It has been observed that viruses can be inactivated by UVA radiation and visible light. The aim of this study is to investigate whether a medium that contains a photosensitizer might have an influence on viral reduction under irradiation by UVA, violet or blue light. Test virus is the bacteriophage PhiX174 in the photosensitizer-free SM buffer and DMEM-F12, which contains the known photosensitizer riboflavin.

**Results:**

The determined PhiX174 D90 doses in SM buffer and DMEM were 36.8 J/cm² and 13.6 J/cm² at 366 nm, 153.6 J/cm² and 129.1 J/cm² at 408 nm and 4988 J/cm² and 2477.1 J/cm² at 455 nm, respectively. It can be concluded that the medium has a large influence on the results. This might be caused by the photosensitizer riboflavin in DMEM-F12. As riboflavin is a key component in many cell culture media, irradiation experiments with viruses in cell culture media should be avoided if the investigation of intrinsical photoinactivation properties of viruses is aimed for.

## Introduction

Since the beginning of the coronavirus pandemic in 2019, the awareness towards viruses has increased and thus also the demand for disinfection in all areas of everyday life and especially in healthcare facilities. The most accepted method is chemical disinfection, e.g. with ethanol [1]. In addition, methods like thermal or UV (ultraviolet) disinfection are also widely applied [2–4]. Especially for UV disinfection, numerous studies have already been published about its effectiveness on viruses [5–9], though it is also harmful to humans [10–13].

As an alternative, disinfection with visible light is already employed in photodynamic therapy [14] and for inactivation of microorganisms, such as bacteria or fungi [15–18]. The mechanism is based on external or endogenous photosensitizers such as flavins and porphyrins, which are stimulated by light absorption. As a result, reactive oxygen species (ROS), such as singlet oxygen or oxygen peroxides, are produced in an oxygen-containing environment. These reactive molecules damage cell structures, like cell membranes or cellular lipids and proteins, by oxidative processes and thus inactivate the cell [19–21].

Even though viruses seem not to be microorganisms [22], studies have already been published, which prove that viruses, such as coronaviruses or influenza viruses, can also be inactivated by irradiation in the blue or violet spectral range [23–27]. Advantage of irradiation in the visible spectral range is that it is much less harmful for human cells than UV radiation [28–30].

However, published irradiation results were performed in different liquid media [23, 31–37] and some of these media contain photosensitizers. The known photosensitizer riboflavin can be found in many culture media but other components like vitamin A, D_2_, D_3_, E and K_1_ might also exhibit photosensitizer properties at least under UV irradiation [32, 38]. However, besides riboflavin, these vitamins are no part of standard media like MEM, DMEM or RPMI. The question arises whether external photosensitizers in the medium have a strong influence on the observed log-reduction doses when irradiated with light in the violet and blue spectral range. For example, the influence of external photosensitizers in the visible spectral range has already been proven for *Escherichia coli* [39, 40]. As Hessling et al. found out by comparing different published results on photoinactivation of viruses by light, the irradiation doses differed by up to a factor of ten depending on the applied medium; moreover, riboflavin may be the most frequently involved external photosensitizer [41].

The resulting wide range of literature values could misrepresent the pure photosensitivity of viruses. Considering the fact that viruses do not have their own metabolism [42] and the hypothesis that they do absorb endogenous photosensitizers from the host [41], the photochemical effect observed in bacteria could also occur in viruses. Since the photosensitivity to visible light has already been verified for *E. coli* [39, 40], a bacteriophage was selected for the study that would infect this bacterium and use it as host. In order to be able to observe possible small effects, a phage was chosen that only has a capsid and no separate envelope, as these are less sensitive to environmental influences [43].

The purpose of this study is to investigate, whether viral photosensitivity depends on the applied medium that contains the viruses. Test virus is the bacteriophage PhiX174 and the irradiation is performed with blue (455 nm) and violet (408 nm) light and UVA radiation (366 nm) for comparison. Media are saline magnesium (SM) buffer (without photosensitizer) and Dulbecco’s Modified Eagle’s Medium DMEM-F12 with a medium riboflavin concentration.

## Materials and methods

**Fig. 1:**
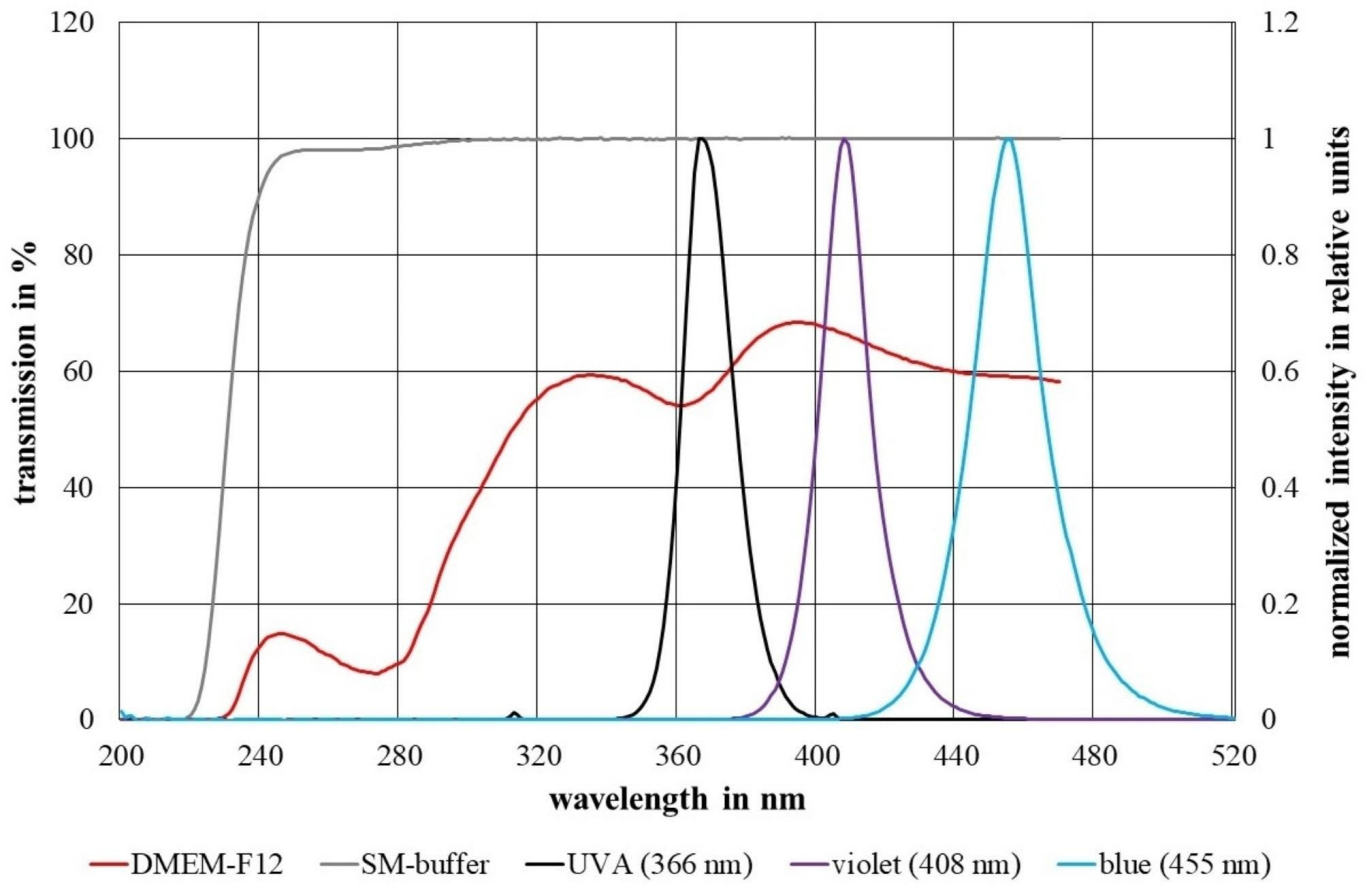
Normalized irradiation spectra and transmission spectra of DMEM-F12 and SM buffer for an optical path length of 10 mm

The bacteriophage PhiX174 (DSM 4497) and its recommended host *E. coli* (DSM 13127) were obtained from DSMZ (German Collection of Microorganisms and Cell Cultures, Braunschweig, Germany). A bacterial colony of *E. coli* was cultured in 3 ml Luria Bertani (LB) medium for 3 h at 37 °C and 170 rpm. Thereafter, the culture exhibited an optical density of 0.15 at 600 nm, which was equivalent to 1 × 10^9^ colony forming units (CFU)/ml. The bacteriophages were enriched in SM buffer as recommended by DSMZ and a stock solution with a titer of 10^8^ viruses / ml was prepared as described by Sambrook and Russel [44]. The SM buffer has a transmission of > 98% for the relevant wavelengths [9]. The transmission of the DMEM-F12 medium (including about 0.2 mg/l riboflavin) used here was measured with a Specord Plus absorption spectrometer of Analytik Jena (Jena, Germany). A 10 mm quartz cuvette filled with distilled water served as a reference. The transmission of both media is illustrated in Fig. 1. The transmittances relevant to this work were 56% @ 366 nm, 67% @ 408 nm and 59% @ 455 nm.

For irradiation in the UVA range, a “*3UV-36 lamp*” from Analytik Jena with a peak emission at 366 nm was selected. In the visible spectral range, high-power LEDs were applied: “*LZ4-40UA00-00U8*” as violet irradiation source and “*LZ4-40B208-0000*” for the blue spectral range, both of Led Engin (San Jose, USA). The irradiances during the experiments, were 1.80 mW/cm² (366 nm, UVA), 17.5 mW/cm² (408 nm, violet light) and 70 mW/cm² (455 nm, blue light).

The phage stock solution was diluted to a concentration of 10^7^ plague forming units per ml (PFU/ml) before each experiment. For the irradiation, a sample of 3 ml was filled into quartz beakers for UVA (366 nm) and in glass beakers for violet and blue light, which had a diameter of 22 mm. The filling height was approximately 10 mm. To prevent heating of the sample and to ensure a constant temperature of 20 °C, the samples were cooled by a water bath type Thermocell of Biozyme Scientific (Hessisch Oldendorf, Germany) during irradiation and controlled frequently. After defined periods of time, 100 µl samples of the irradiated phage suspension were taken and irradiation was continued. Each time, three samples were taken and three independent series of experiments were performed. Phages dissolved in SM buffer or DMEM were each diluted to 10^− 6^ by dilution series (900 µl SM buffer + 100 µl sample). 100 µl of each phage sample was mixed with 100 µl of bacterial suspension diluted 1/10 with phosphate buffered saline. After an incubation period of 10 min, the phage-bacteria suspension was mixed with soft agar and poured on to LB agar plates using the “double agar layering technique” referred to by DSMZ [24, 45]. Soft agar was based on LB medium but contained only 6 g/L of agar, resulting in low viscosity. It was not supposed to fall below or rise above a temperature of 50 °C, otherwise the soft agar would solidify or the phages or bacteria would start to denature. Subsequently, the agar plates were placed in a 37 °C incubator to incubate the bacteria with the phages for 4–6 h. After reaching the incubation time, the plaques in the bacterial lawn were counted. By comparing the PFU before and after irradiation, the achieved reduction of phages was depicted. Average log-reduction doses were calculated by the achieved reduction at the highest applied dose. The D90 dose was determined on the basis of the first reduction of 90% or higher.

## Results

**Fig. 2:**
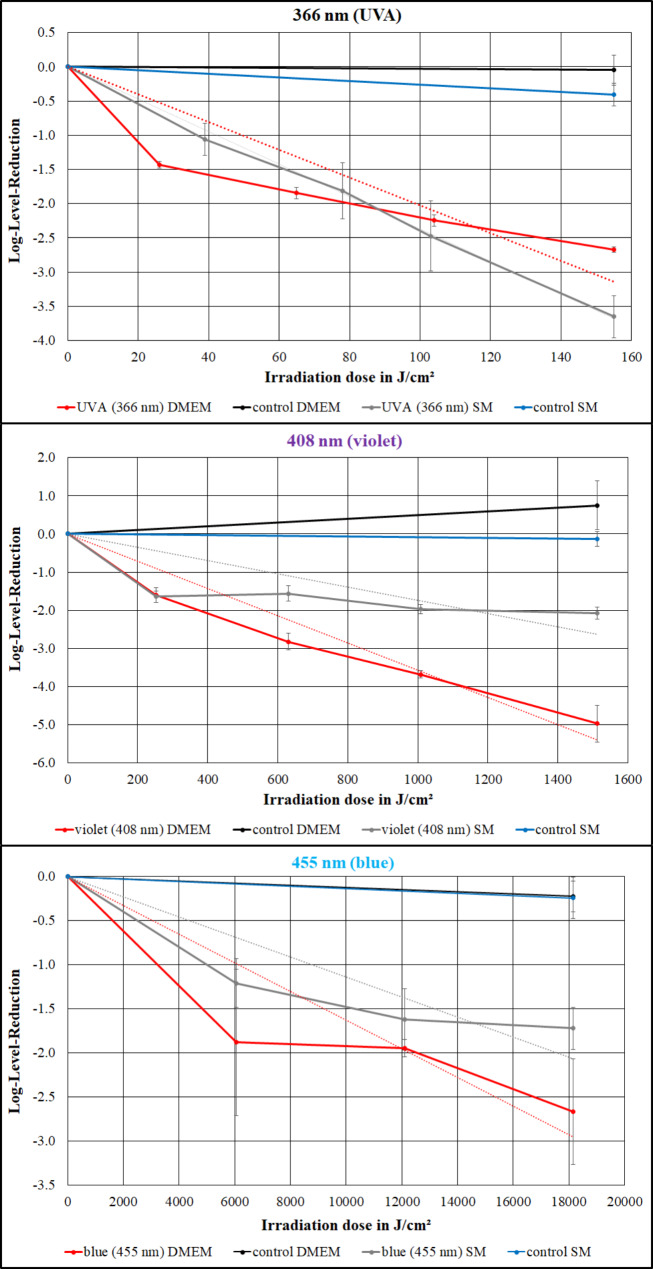
Irradiation of the phage PhiX174 at 366 nm (top), 408 nm (middle) and 455 nm (below). The reduction in DMEM is plotted in red and for SM in grey. The corresponding unirradiated controls are black (DMEM) and blue (SM). The error bars give the standard deviation of the triplicates

A phage reduction was observed in both media, with large differences between the different wavelengths. The results are illustrated in Fig. 2. The course of the reduction deviates more or less from an exponential course, which would be a straight line in the semi-logarithmic representations. Detailed results are listed below.

### 366 nm irradiation (UVA)

The 366 nm (UVA) inactivation experiment lasted 24 h, which corresponded to a total irradiation dose of 155 J/cm². An average log-reduction dose (total irradiation dose / total log-reduction) of 58.2 J/cm² in DMEM and 42.5 J/cm² in SM buffer was determined for one log level reduction of PhiX174. The corresponding D90 doses are 18.2 J/cm² in DMEM and 36.8 J/cm² in SM buffer.

### 408 nm irradiation (violet light)

The 408 nm (violet) inactivation experiment lasted 24 h, which corresponded to a total irradiation dose of 1512 J/cm². An average log-reduction dose of 303.7 J/cm² in DMEM and 729.8 J/cm² in SM buffer was determined for one log level reduction of PhiX174. The corresponding D90 doses are 157.5 J/cm² in DMEM and 153.6 J/cm² in SM buffer.

### 455 nm irradiation (blue light)

The 455 nm (blue) inactivation experiment lasted 72 h, which corresponded to a total irradiation dose of 18,144 J/cm². An average log-reduction dose of 6796.3 J/cm² in DMEM and 10,506 J/cm² in SM buffer was determined for one log level reduction of PhiX174. The corresponding D90 doses are 3217 J/cm² in DMEM and 4998 J/cm² in SM buffer.

## Discussion

The low DMEM transmission illustrated in Fig. 1 complicates a quantitative comparison of PhiX174 photosensitivities between both media. Therefore, as a more realistic measure of the actual applied average irradiation dose inside DMEM, the irradiation intensity and irradiation behind a 5 mm DMEM layer (half the sample height) is calculated by the Beer-Lambert law.

The corrected log-reduction doses and D90 doses for PhiX174 in DMEM and the corresponding unmodified SM buffer doses are listed in Table 1.

**Table 1:** Corrected log-reduction doses and D90 doses for DMEM and corresponding unmodified doses for SM buffer

wavelength [nm]	medium	average log-reduction dose [J/cm^2^]	average log-reduction dose ratio	D90 dose [J/cm^2^]	D90 dose ratio
366 UVA	DMEM	43.6	0.97	13.6	2.71
	SM	42.5		36.8	
408 violet	DMEM	249	2.93	129.1	1.19
	SM	729.8		153.6	
455 blue	DMEM	5233.1	2.00	2477	2.02
	SM	10,506		4998	

The higher the wavelength the higher the average log-reduction doses and D90 doses with a difference of about two orders of magnitude between 366 and 455 nm. The determined D90 doses are always lower in DMEM. This higher photosensitivity might be caused by the riboflavin within the DMEM as riboflavin is a known photosensitizer with strong absorption in the UVA and violet and blue spectral range [46–48]. As DMEM-F12 also contains other components besides riboflavin, including other vitamins, it is open whether the observed photoinactivation is solely caused by riboflavin. However, more important is the fact that there are actually photosensitizers in the medium and they influence the photoinactivation results.

This difference between DMEM and SM buffer is also observed for the average log-reduction doses for 408 and 455 nm irradiation, but not at 366 nm. For this wavelength, the average log-reduction doses are virtually identical, although the virus reduction at small doses is evidently stronger in DMEM. The latter is well illustrated by the non-linear or non-exponential curve of virus reduction in DMEM in Fig. 2, which exhibits a substantially reduced photosensitivity after higher irradiation doses.

Since riboflavin is known to be not photostable [46–50], it could be that riboflavin is no longer present at higher doses, reducing the difference between the media.

However, quantitative testing of the hypothesis that the differences between virus inactivation in DMEM and SM buffer are caused only or predominantly by riboflavin in the medium is difficult. First, the PhiX174 photoinactivation mechanism is not understood even in SM buffer, and second, riboflavin photochemistry is complex. Photolysis of riboflavin leads predominantly to the formation of lumichrome, which can also act as a photosensitizer but exhibits different absorption properties than riboflavin [47–49].

## Conclusions

Irradiation of PhiX174 at 366 nm (UVA), 408 nm (violet) or 455 nm (blue) lead to virus inactivation by all wavelengths and in both media. The PhiX174 photosensitivities, expressed as average log-reduction doses or D90 doses, differ by approximately two orders of magnitude between 366 and 455 nm.

There is also a noticeable difference in the photosensitivities between PhiX174 in DMEM and SM buffer, especially if the optical transmission of DMEM is considered. This might be caused by riboflavin in DMEM, which could act as external photosensitizer.

Therefore, if intrinsical photosensitivities of viruses are investigated, external photosensitizers that are commonly found in cell culture media, should be avoided.

### Limitations

Here, we examined only a single phage to determine whether photoinactivation was dependent on the medium and no additional viruses. However, PhiX174 is the first virus we have ever studied in this context, and already in this first virus we found a medium dependency. We suspect that this is also true for other viruses, or if it is declared that a virus photoinactivation does not depend on the medium, this must be proven.

## Data Availability

The datasets used and/or analysed during the current study are available from the corresponding author on reasonable request.

## Abbreviations

LED: light emitting diode
UVA: Ultraviolet radiation in the spectral range 315 to 400 nm
DMEM: Dulbecco’s Modified Eagle’s Medium
SM buffer: saline magnesium buffer
PBS: phosphate buffered saline

## References

[1] Kratzel A, Todt D, V’kovski P, Steiner S, Gultom M, Thao TTN (2020). Inactivation of severe Acute Respiratory Syndrome Coronavirus 2 by WHO-Recommended Hand Rub formulations and alcohols. Emerg Infect Dis.

[2] Kariwa H, Fujii N, Takashima I (2006). Inactivation of SARS coronavirus by means of povidone-iodine, physical conditions and chemical reagents. Dermatology.

[3] Kampf G, Voss A, Scheithauer S (2020). Inactivation of coronaviruses by heat. J Hosp Infect.

[4] Heßling M, Hönes K, Vatter P, Lingenfelder C (2020). Ultraviolet irradiation doses for coronavirus inactivation - review and analysis of coronavirus photoinactivation studies. GMS Hyg Infect Control.

[5] Inagaki H, Saito A, Sugiyama H, Okabayashi T, Fujimoto S (2020). Rapid inactivation of SARS-CoV-2 with deep-UV LED irradiation. Emerg Microbes Infect.

[6] Jensen MM (1964). Iinactivation of airborne viruses by ultraviolet irradiation. Appl Microbiol.

[7] Storm N, McKay LGA, Downs SN, Johnson RI, Birru D, de Samber M (2020). Rapid and complete inactivation of SARS-CoV-2 by ultraviolet-C irradiation. Sci Rep.

[8] Weyersberg L, Klemens E, Buehler J, Vatter P, Hessling M (2022). UVC, UVB and UVA susceptibility of Phi6 and its suitability as a SARS-CoV-2 surrogate. AIMS Microbiol.

[9] Weyersberg L, Sommerfeld F, Vatter P, Hessling M (2023). UV radiation sensitivity of bacteriophage PhiX174 - A potential surrogate for SARS-CoV-2 in terms of radiation inactivation. AIMS Microbiol.

[10] D’Orazio J, Jarrett S, Amaro-Ortiz A, Scott T (2013). UV radiation and the skin. Int J Mol Sci.

[11] Matsumura Y, Ananthaswamy HN (2004). Toxic effects of ultraviolet radiation on the skin. Toxicol Appl Pharmacol.

[12] Ravanat JL, Douki T, Cadet J (2001). Direct and indirect effects of UV radiation on DNA and its components. J Photochem Photobiol B.

[13] Roy S (2017). Impact of UV Radiation on Genome Stability and Human Health. Adv Exp Med Biol.

[14] Dougherty TJ, Gomer CJ, Henderson BW, Jori G, Kessel D, Korbelik M (1998). Photodynamic therapy. J Natl Cancer Inst.

[15] Hoenes K, Bauer R, Meurle T, Spellerberg B, Hessling M (2020). Inactivation effect of Violet and Blue Light on ESKAPE pathogens and closely related non-pathogenic bacterial species - A Promising Tool against antibiotic-sensitive and antibiotic-resistant microorganisms. Front Microbiol.

[16] Cieplik F, Späth A, Leibl C, Gollmer A, Regensburger J, Tabenski L (2014). Blue light kills Aggregatibacter actinomycetemcomitans due to its endogenous photosensitizers. Clin Oral Investig.

[17] Maclean M, MacGregor SJ, Anderson JG, Woolsey G (2009). Inactivation of bacterial pathogens following exposure to light from a 405-nanometer light-emitting diode array. Appl Environ Microbiol.

[18] Pileggi G, Wataha JC, Girard M, Grad I, Schrenzel J, Lange N, Bouillaguet S (2013). Blue light-mediated inactivation of Enterococcus faecalis in vitro. Photodiagnosis Photodyn Ther.

[19] Plavskii VY, Mikulich AV, Tretyakova AI, Leusenka IA, Plavskaya LG, Kazyuchits OA (2018). Porphyrins and flavins as endogenous acceptors of optical radiation of blue spectral region determining photoinactivation of microbial cells. J Photochem Photobiol B.

[20] Lui GY, Roser D, Corkish R, Ashbolt NJ, Stuetz R (2016). Point-of-use water disinfection using ultraviolet and visible light-emitting diodes. Sci Total Environ.

[21] Cunningham ML, Krinsky NI, Giovanazzi SM, Peak MJ (1985). Superoxide anion is generated from cellular metabolites by solar radiation and its components. J Free Radic Biol Med.

[22] Michel H-W (2022). Die Welt Der Viren Und Mikroorganismen.

[23] Vatter P, Hoenes K, Hessling M (2021). Photoinactivation of the Coronavirus Surrogate phi6 by visible light. Photochem Photobiol.

[24] Vatter P, Hoenes K, Hessling M (2021). Blue light inactivation of the enveloped RNA virus Phi6. BMC Res Notes.

[25] Lau B, Becher D, Hessling M (2021). High intensity Violet Light (405 nm) inactivates coronaviruses in phosphate buffered saline (PBS) and on surfaces. Photonics.

[26] Rathnasinghe R, Jangra S, Miorin L, Schotsaert M, Yahnke C, Garcίa-Sastre A (2021). The virucidal effects of 405 nm visible light on SARS-CoV-2 and Influenza a virus. Sci Rep.

[27] Biasin M, Strizzi S, Bianco A, Macchi A, Utyro O, Pareschi G (2022). UV and violet light can neutralize SARS-CoV-2 infectivity. J Photochem Photobiol.

[28] Liebmann J, Born M, Kolb-Bachofen V (2010). Blue-light irradiation regulates proliferation and differentiation in human skin cells. J Invest Dermatol.

[29] Makdoumi K, Hedin M, Bäckman A (2019). Different photodynamic effects of blue light with and without riboflavin on methicillin-resistant Staphylococcus aureus (MRSA) and human keratinocytes in vitro. Lasers Med Sci.

[30] Opländer C, Hidding S, Werners FB, Born M, Pallua N, Suschek CV (2011). Effects of blue light irradiation on human dermal fibroblasts. J Photochem Photobiol B.

[31] Time dose reciprocity in UV disinfection of water (1998). Water Sci Technol.

[32] Appleyard G (1967). The photosensitivity of Semliki Forest and other viruses. J Gen Virol.

[33] Battigelli DA, Sobsey MD, Lobe DC (1993). The inactivation of Hepatitis a Virus and other Model viruses by UV irradiation. Water Sci Technol.

[34] Cutchins EC, Dayhuff TR (1962). Photoinactivation of Measles virus. Virology.

[35] Lo C-W, Matsuura R, Iimura K, Wada S, Shinjo A, Benno Y (2021). UVC disinfects SARS-CoV-2 by induction of viral genome damage without apparent effects on viral morphology and proteins. Sci Rep.

[36] Minamikawa T, Koma T, Suzuki A, Mizuno T, Nagamatsu K, Arimochi H (2021). Quantitative evaluation of SARS-CoV-2 inactivation using a deep ultraviolet light-emitting diode. Sci Rep.

[37] Sommer R, Pribil W, Appelt S, Gehringer P, Eschweiler H, Leth H (2001). Inactivation of bacteriophages in water by means of non-ionizing (UV-253.7 nm) and ionizing (gamma) radiation: a comparative approach. Water Res.

[38] Knak A, Regensburger J, Maisch T, Bäumler W (2014). Exposure of vitamins to UVB and UVA radiation generates singlet oxygen. Photochem Photobiol Sci.

[39] Fyrestam J (2018). Porphyrins and heme in microorganisms: porphyrin content and its relation to phototherapy and antimicrobial treatments in vivo and in vitro.

[40] Liang J-Y, Yuann J-MP, Cheng C-W, Jian H-L, Lin C-C, Chen L-Y (2013). Blue light induced free radicals from Riboflavin on E. Coli DNA damage. J Photochem Photobiol B.

[41] Hessling M, Lau B, Vatter P (2022). Review of Virus inactivation by visible light. Photonics.

[42] Golais F, Hollý J, Vítkovská J (2013). Coevolution of bacteria and their viruses. Folia Microbiol (Praha).

[43] Deutschland, German BioSafety (2011). Biologische Gefahren in Deutschland: Proceedings; Kongressbericht Der German BioSafety 2005 ; wissenschaftliche Konferenz Mit Angeschlossener Fachmesse Vom 13. Bis 15. September 2005 in Stuttgart.

[44] Molecular Cloning, Russell DW (2001). Q Rev Biol.

[45] Cormier J, Janes M (2014). A double layer plaque assay using spread plate technique for enumeration of bacteriophage MS2. J Virol Methods.

[46] Insińska-Rak M, Sikorski M (2014). Riboflavin interactions with oxygen-a survey from the photochemical perspective. Chemistry.

[47] Huang R, Kim HJ, Min DB (2006). Photosensitizing effect of Riboflavin, lumiflavin, and lumichrome on the generation of volatiles in soy milk. J Agric Food Chem.

[48] Oster G, Bellin JS, Holmstrom B (1962). Photochemistry of Riboflavin. Experientia.

[49] Ahmad I, Fasihullah Q, Noor A, Ansari IA, Ali QNM (2004). Photolysis of Riboflavin in aqueous solution: a kinetic study. Int J Pharm.

[50] Sheraz MA, Kazi SH, Ahmed S, Anwar Z, Ahmad I (2014). Photo, thermal and chemical degradation of Riboflavin. Beilstein J Org Chem.

